# Coordination of the Co^2+^ and Ni^2+^ Ions in Tf_2_N^–^ Based Ionic Liquids: A Combined X-ray Absorption and Molecular Dynamics Study

**DOI:** 10.1021/acs.jpcb.1c03395

**Published:** 2021-06-10

**Authors:** Matteo Busato, Andrea Lapi, Paola D’Angelo, Andrea Melchior

**Affiliations:** † Dipartimento di Chimica, Università di Roma “La Sapienza”, Piazzale Aldo Moro 5, 00185 Roma, Italy; ‡ DPIA, Laboratorio di Scienze e Tecnologie Chimiche, Università di Udine, Via del Cotonificio 108, 33100 Udine, Italy

## Abstract

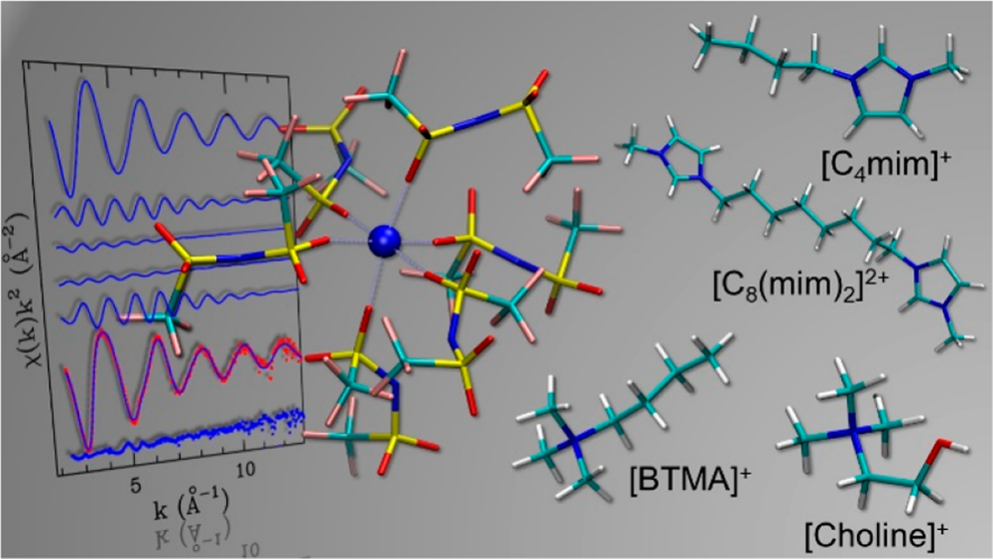

Molecular dynamics (MD) simulations and X-ray absorption spectroscopy (XAS) have been combined to study the coordination of the Co^2+^ and Ni^2+^ ions in ionic liquids (ILs) based on the bis(trifluoromethylsulfonyl)imide ([Tf_2_N]^−^) anion and having different organic cations, namely, 1-butyl-3-methylimidazolium ([C_4_mim]^+^), 1,8-bis(3-methylimidazolium-1-yl)octane ([C_8_(mim)_2_]^2+^), *N*,*N*,*N*-trimethyl-*N*-(2-hydroxyethyl)ammonium ([choline]^+^), and butyltrimethylammonium ([BTMA]^+^). Co and Ni K-edge XAS data have been collected on 0.1 mol L^–1^ Co(Tf_2_N)_2_ and Ni(Tf_2_N)_2_ solutions and on the metallic salts. MD simulations have been carried out to obtain structural information on the metal ion coordination. The analysis of the extended X-ray absorption fine structure (EXAFS) spectra of the solutions has been carried out based on the atomistic description provided by MD, and the studied ILs have been found to be able to dissolve both the Co(Tf_2_N)_2_ and Ni(Tf_2_N)_2_ salts giving rise to a different structural arrangement around the metal ions as compared to the solid state. The combined EXAFS and MD results showed that the Co^2+^ and Ni^2+^ ions are surrounded by a first solvation shell formed by six [Tf_2_N]^−^ anions, each coordinating in a monodentate fashion by means of the oxygen atoms. The nature of the IL organic cation has little or no influence on the overall spatial arrangement of the [Tf_2_N]^−^ anions, so that stable octahedral complexes of the type [M(Tf_2_N)_6_]^4–^ (M = Co, Ni) have been observed in all the investigated ILs.

## 1 Introduction

The extraction and separation of the Co^2+^ and Ni^2+^ ions from complex aqueous matrixes have recently received much attention for the recovering of these metals from acidic aqueous solutions originating from mining activity^1,2^ and for their recycling from devices that are at their end-of-life.^3^ In particular, the recycling of cobalt and nickel present in the cathodes of lithium-ion batteries (lithium–nickel–manganese–cobalt oxide, NMC) has assumed a growing importance in the last years in order to meet a global demand that is increasing due to the expansion of electric mobility and to the uncertain access to primary sources.^4,5^ Current hydrometallurgical processes for the recovery of the Co^2+^ and Ni^2+^ ions from aqueous solutions present some interesting advantages (low energy consumption, process flexibility, higher purity...) but require the use of significant amounts of toxic volatile organic compounds (*e*.*g*., liquid hydrocarbons) in the solvent extraction stages and of multiple extracting ligands to separate the different metals present in the leachates.^6^

In the last two decades, ionic liquids (ILs) attracted much attention as a more sustainable alternative to traditional organic solvents owing to their practically negligible vapor pressure, non-flammability, thermal stability, wide electrochemical windows, good solvation ability for both neutral and charged species, and low toxicity.^7^ Due to these features, ILs emerged as new media for a variety of applications, such as electroplating, batteries, solar cells, corrosion protection, catalysis, food and pharmaceutical applications.^8−14^ Moreover, ILs have been found to be suitable as the hydrophobic receiving phase for the separation of target metal ions from aqueous solutions.^15−20^ Also, thanks to the wide electrochemical windows accessible, they can serve as suitable media for metal electrodepositions.^11,21−25^

The use of ILs in the simultaneous recovery of cobalt and nickel from aqueous solutions has been investigated in several recent studies aimed at developing more sustainable separation processes either using the “neat” ILs or in the presence of auxiliary extracting ligands.^6,26−30^

In this framework, the knowledge of the nature of the interactions between the dissolved metal ions and the IL anions is key information to understand the solvation/desolvation phenomena^31−33^ and the thermodynamics of complex formation and speciation. For example, the thermodynamic parameters obtained in some studies on the formation of Ni^2+^ complexes with dimethyl sulfoxide, methanol, and acetonitrile in 1-alkyl-3-methylimidazolium ([C_*n*_mim]) bis(trifluoromethylsulfonyl)imide ([Tf_2_N]^−^) ILs (where C_*n*_ is the length of the alkyl chain, in this work *n* = 2, 8)^34,35^ and with nitrate in [C_4_mim][Tf_2_N]^36^ could be reinterpreted if the coordination geometry of the starting solvated metal ions was established. However, the structural characterization of metal ion solvation complexes in disordered liquid samples is known to be a difficult task.^37−43^ In this respect, due to its unique short-range sensitivity and chemical selectivity, the X-ray absorption spectroscopy (XAS) technique is an ideal candidate to provide accurate structural information on the local arrangement of the solvent molecules around a metal ion.^44,45^ In particular, the combination between XAS and molecular dynamics (MD) simulations provides an invaluable tool for the structural characterization of metal ion solutions in ILs.^36,46−48^ Through the synergic use of the XAS and MD techniques, one can also check the validity of the level of theory employed in the simulations directly on the experimental evidence, while having at the same time a reliable structural model to be used in the analysis of the experimental data.

**Figure 1:**
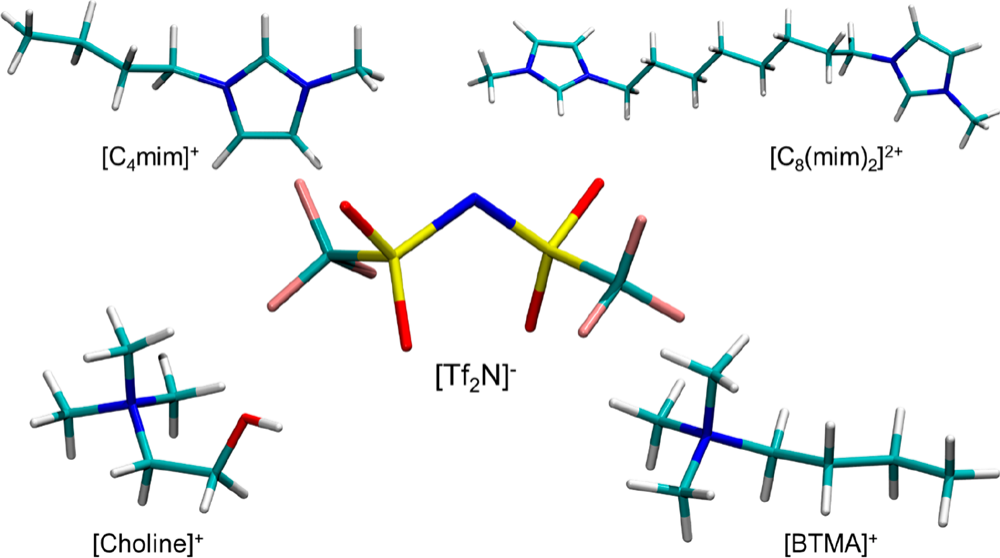
Molecular structures of the IL cations and anions investigated in this work: 1-butyl-3-methylimidazolium ([C_4_mim]^+^), 1,8-bis(3-methylimidazolium-1-yl)octane ([C_8_(mim)_2_]^2+^), *N*,*N*,*N*-trimethyl-*N*-(2-hydroxyethyl)ammonium ([choline]^+^), butyltrimethylammonium ([BTMA]^+^) cations within the bis(trifluoromethylsulfonyl)imide ([Tf_2_N]^−^) anion.

In the present work, we employ a combined approach between XAS and MD to perform a structural characterization of the solvation complexes formed by the Co^2+^ and Ni^2+^ ions in ILs based on the [Tf_2_N]^−^ anion and having different organic cations, namely, [C_4_mim]^+^, 1,8-bis(3-methylimidazolium-1-yl)octane ([C_8_(mim)_2_]^2+^), *N*,*N*,*N*-trimethyl-*N*-(2-hydroxyethyl)ammonium ([choline]^+^), and butyltrimethylammonium ([BTMA]^+^). The molecular structures of the IL cations and of the anion investigated in this work are shown in Figure 1. By comparing the properties of the different systems, we were able to assess whether the nature of the IL organic cation has influence on the structural arrangement of the [Tf_2_N]^−^ anion coordinating the Co^2+^ and Ni^2+^ ions. The obtained structural insights will aid the employment of metal ion solutions in ILs as advanced processing media.

## 2 Methods

### 2.1 Molecular Dynamics Simulations

MD simulations have been carried out on Co^2+^ and Ni^2+^ solutions in [C_4_mim][Tf_2_N], [C_8_(mim)_2_][Tf_2_N]_2_, [choline][Tf_2_N], and [BTMA][Tf_2_N]. The ILs were represented by the all-atom nonpolarizable force field developed by Canongia Lopes and Padua.^49−52^ The Lennard–Jones (LJ) potential was used for the van der Waals (vdW) part and the Lorentz–Berthelot combination rules were employed to build the cross-terms. LJ parameters for the Co^2+^ ion were taken from Merz and Li “IOD” set,^53^ while for the Ni^2+^ ion the LJ parameters developed by Fulton et al.^54^ were employed. The composition of the simulated systems is reported in Table S1. The number of metal cations and IL ion couples were chosen in order to reproduce the 0.1 mol L^–1^ concentration of the Co(Tf_2_N)_2_ and Ni(Tf_2_N)_2_ solutions employed for the XAS measurements and to match the pure IL experimental densities.^55−58^

Simulation boxes were first equilibrated in NVT conditions by gradually bringing each system from 298 to 700 K, keeping it at high temperature for 10 ns and gradually cooling down to 298 K. High-temperature equilibrations were previously observed to be mandatory for viscous liquids like ILs to improve the system dynamics.^46−48^ Production runs for data collection were performed for 100 ns in NVT conditions at 298 K with configurations saved every 100 steps. A cutoff radius of 12 Å was employed for all the nonbonded interactions with a switching function from 10 to 12 Å for the vdW part, while long-range electrostatic effects were calculated by the particle mesh Ewald (PME) method.^59,60^ A 1 fs time step was employed in all the simulations, and the temperature was kept constant with the thermostat implicitly handled by the stochastic dynamics leapfrog integrator with a coupling constant of 0.5 ps.^61^

The structural properties of the solutions have been characterized by calculating the radial (*g*(*r*)s) and combined distribution functions (CDFs).^62^ To have a quantitative description of the coordination around the Co^2+^ and Ni^2+^ ions, M–O, M–S, and M–N (M = Co, Ni) *g*(*r*)s were modeled with Γ-like functions depending on four parameters: the average distance *R*, the coordination number *N*, the standard deviation σ^2^, and the asymmetry index β. This function is defined in a wide interval of positive and negative asymmetry values and falls in the Gaussian limit for β → 0.^46^

Simulations were performed with the Gromacs 2019.6 program.^63^ Initial configurations were built by randomizing the atomic positions with the PACKMOL package,^64^ and the VMD 1.9.3 software^65^ was used for trajectories visualization. CDFs were calculated with an in-house written code.^62^

### 2.2 X-ray Absorption Experiments

[C_4_mim][Tf_2_N] was purchased from Sigma-Aldrich, while [choline][Tf_2_N] and [BTMA][Tf_2_N] from Iolitec GmbH, all with a stated purity >99%. [C_8_(mim)_2_][Tf_2_N]_2_ was synthesized with the procedure reported by Mandai et al.^66^ The Co(Tf_2_N)_2_ and Ni(Tf_2_N)_2_ salts were synthesized following the procedure reported by Earle et al.^67^ The eight 0.1 mol L^–1^ solutions of Co(Tf_2_N)_2_ and Ni(Tf_2_N)_2_ in [C_4_mim][Tf_2_N], [C_8_(mim)_2_][Tf_2_N]_2_, [choline][Tf_2_N], and [BTMA][Tf_2_N] were prepared by adding stoichiometric amounts of the correspondent salt into the ILs. The resulting solutions were sonicated for 10 min and then dried under vacuum for 36 h.

Co and Ni K-edge XAS spectra of the IL solutions and of solid Co(Tf_2_N)_2_ and Ni(Tf_2_N)_2_ were acquired in transmission mode at the Elettra-Sincrotrone Trieste 11.1 beamline.^68^ The liquid samples were inserted in cells with Kapton windows and kept under N_2_ flux during collection to prevent contact with the air moisture. Solid Co(Tf_2_N)_2_ and Ni(Tf_2_N)_2_ were carefully grained in an agate mortar together with boron nitride as diluting and put in aluminum frames (1.5 mm thick) covered with a Mylar tape. The beamline was equipped with a Si(111) double crystal monochromator, and during the measurements the storage ring was operating at 2 GeV and the beam current was 200 mA. For each sample, at least three spectra were collected and averaged.

### 2.3 EXAFS Data Analysis

**Figure 2:**
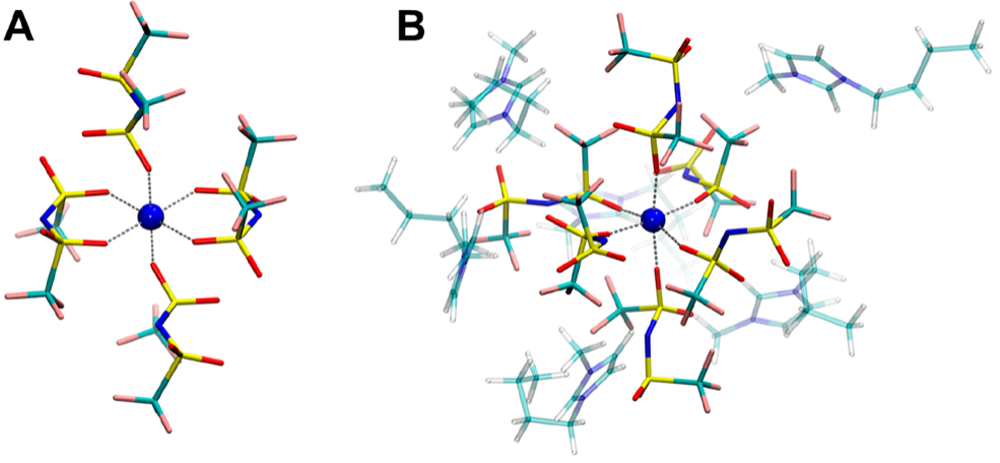
[Co(Tf_2_N)_4_]^2–^ unit in the crystallographic structure^72^ of [C_1_C_4_Pyr]_2_[Co(Tf_2_N)_4_] (C_1_C_4_Pyr = 1-butyl-1-methylpyrrolidinium) (panel A) and a representative snapshot of the [Co(Tf_2_N)_6_]^4–^ unit obtained from the MD simulation of Co(Tf_2_N)_2_ in [C_4_mim][Tf_2_N] (panel B; opaque, Co^2+^ and [Tf_2_N]^−^, transparent, second shell [C_4_mim]^+^).^48^

The GNXAS program^69,70^ was used to carry out the analysis of the EXAFS part of the absorption spectra. Muffin-tin potentials and advanced models for the exchange-correlation self-energy (Hedin–Lundqvist)^71^ were employed to calculate the amplitude and phase shifts. Inelastic losses of the photoelectron in the final state have been taken into account by the complex potential. Details about the EXAFS analysis of the Co(Tf_2_N)_2_ salt can be found in ref (48), while the octahedral [Co(Tf_2_N)_4_]^2–^ unit present in the crystallographic structure is shown in Figure 2A.^72^ The EXAFS analysis of the crystalline Ni(Tf_2_N)_2_ spectrum has been carried out using the same structure as the starting model. To this purpose, the Ni–O single scattering (SS) signal related to the six coordinating oxygen atoms, together with the Ni–S and Ni–N signals associated with the four [Tf_2_N]^−^ anions coordinated with the metal cation has been calculated. In addition, the multiple scattering (MS) contribution associated with the Ni–O–S three-body configurations has been found to provide a detectable amplitude. Note that a Ni–O–S configuration with a bond angle of 155° is found both in the mono- and bidentate ligands, giving rise to a MS signal with a total multiplicity of six. The O–S distance of the [Tf_2_N]^−^ anion has been kept fixed to that found in the crystallographic structure (1.47 Å). The MS signal connected with the collinear O–Ni–O distributions has been also considered in the calculations.

The EXAFS analyses of the Co(Tf_2_N)_2_ and Ni(Tf_2_N)_2_ IL solutions have been carried out starting from the structural results obtained from the MD simulations. Theoretical signals for the M–O, M–N, and M–S two-body distributions have been calculated using the structural parameters determined from the MD *g*(*r*)s. In the IL solutions, the [Tf_2_N]^−^ ligands assume a quasi-linear geometry around the metal ions with M–O–S angles of about 180°. The MS contributions related to the O–M–O collinear configurations have been also included in the fitting procedures. Least-squares minimizations have been performed on the raw data directly, and no preliminary Fourier filtering and background subtraction were applied. Nonstructural parameters were also optimized, namely, the K-edge ionization energy *E*_0_ and the energy position and amplitude of the KM_1_ and KM_2,3_ double-electron excitation channels. The inclusion of the double-electron excitations allowed us to keep the *S*_0_^2^ amplitude reduction factor constrained between 0.95 and 0.99.

## 3 Results and Discussion

### 3.1 Molecular Dynamics Results

**Figure 3:**
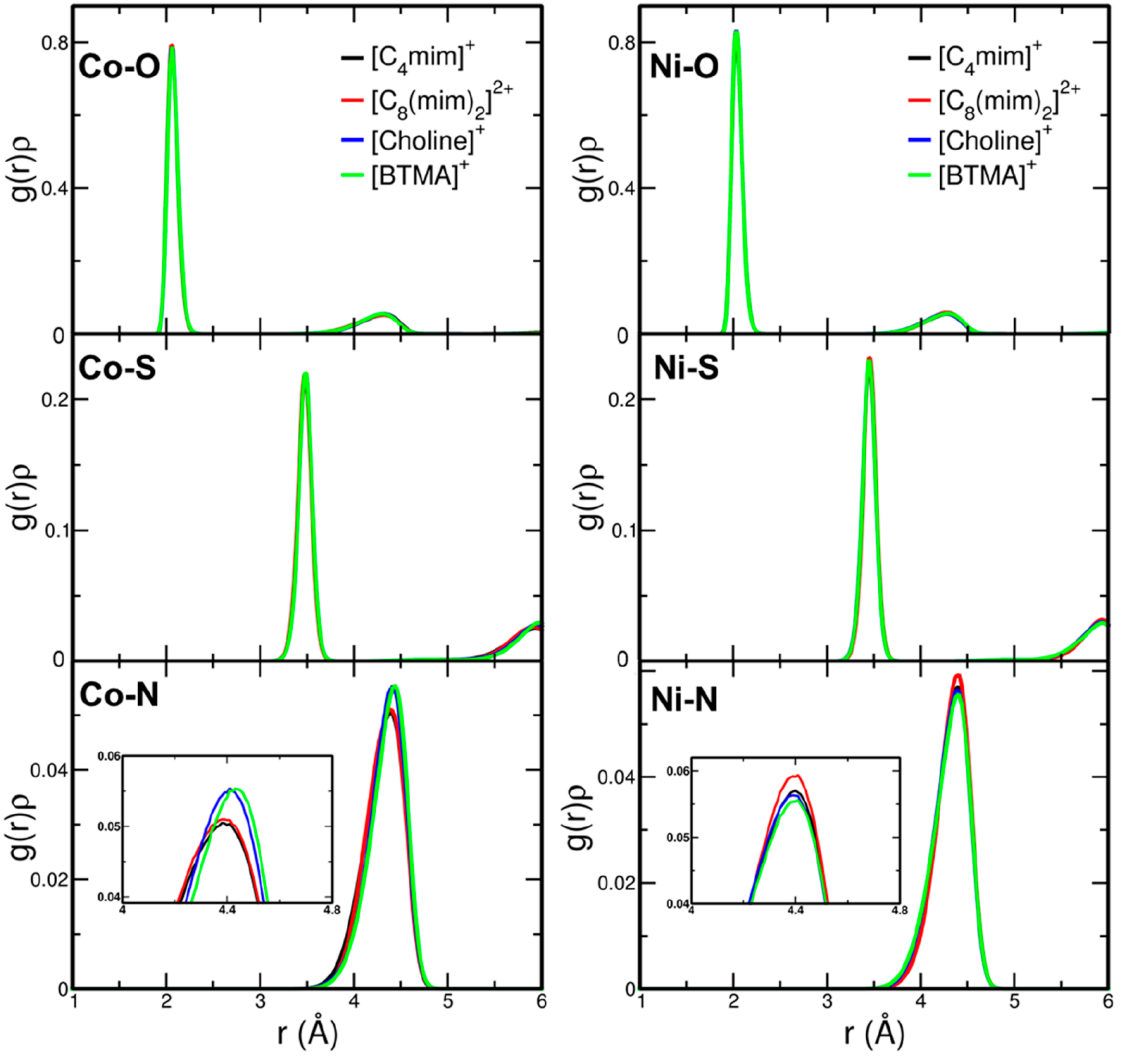
Radial distribution functions multiplied by the numerical densities of the observed atoms, *g*(*r*)ρs, calculated for the M–O, M–S, and M–N (M = Co, Ni) pairs from the MD simulations of the Co(Tf_2_N)_2_ and Ni(Tf_2_N)_2_ solutions in ILs carrying the [Tf_2_N]^−^ anion with different organic cations: [C_4_mim]^+^ (black lines), [C_8_(mim)_2_]^2+^ (red lines), [choline]^+^ (blue lines), and [BTMA]^+^ (green lines). In the case of the M–N pair distributions, insets are also shown to highlight the maxima of the peaks.

The M–O, M–S, and M–N *g*(*r*)s obtained from the MD simulations (Figure 3) were calculated to characterize the local structure around the metal ions. The radial distributions have been multiplied by the number densities of the observed atoms (ρ), since the mere *g*(*r*) can be misleading when comparing systems with different densities.^46,73−75^

The *g*(*r*)s have been modeled with Γ-like functions as previously described to obtain a more quantitative description of the metal ions local coordination. The results of this analysis for Ni(Tf_2_N)_2_ in [C_4_mim][Tf_2_N] are reported in Figure S1, while the structural parameters obtained for all the studied systems are listed in Table 1. As can be observed in Figure 3, the existence of well-defined first solvation shells around the metal ions is testified by the sharp main peaks of the Co–O and Ni–O *g*(*r*)s and by the wide plateaus in the distance range between 2.5 and 3.5 Å. Fits of the M–O *g*(*r*) first peaks with Γ-functions provided a coordination number of six for both the Co^2+^ and Ni^2+^ ions in all the studied ILs (Table 1). The average M–O bond distances obtained from the MD simulations are 2.07 and 2.04–2.05 Å for the Co^2+^ and Ni^2+^ ions, respectively, for all the investigated ILs. As far as the M–S *g*(*r*)s are concerned (Figure 3), well-defined first peaks are present with a coordination number of six (Table 1). Conversely, the M–N *g*(*r*)s are wider (Figure 3) and two asymmetric functions (M–N_1_ and M–N_2_) had to be used to properly reproduce the first peak (Figure S1C). Nevertheless, the sum of the nitrogen atoms of the M–N_1_ and M–N_2_ distribution functions provides a total Ni–N coordination number of six, thus supporting the occurrence of the [M(Tf_2_N)_6_]^4–^ species in solution. The presence of two distinguished M–N distributions has been previously associated with a reorganization of the first solvation sphere due to the steric hindrance connected with the presence of six [Tf_2_N]^−^ anions around the metal ion, so that a certain amount of bis(trifluoromethylsulfonyl)imide ligands are arranged in a slightly different way with respect to the remaining ones.^48^ Note that the well-defined *g*(*r*)s first peaks (Figure 3), along with the integer coordination numbers (Table 1), testify that stable solvation complexes of the [M(Tf_2_N)_6_]^4–^ kind are obtained through the analyzed MD trajectories. In addition, the M–O, M–S, and M–N *g*(*r*)s are nearly superimposable for the same metal ion in all the investigated ILs (Figure 3), showing that the IL organic cation has no influence on the overall spatial arrangement of the [Tf_2_N]^−^ anion coordination.

**Table 1:** Coordination Number *N*, Average Distance *R*, Debye–Waller Factor σ^2^, and Asymmetry Parameter β of the M–O, M–S, and M–N (M = Co, Ni) *g*(*r*)s as Obtained by Modeling the First Peaks with Γ-Like Functions^a^

		M = Co^2+^				M = Ni^2+^			
*g*(*r*)		[C_4_mim]^+^^b^	[C_8_(mim)_2_]^2+^	[choline]^+^	[BTMA]^+^	[C_4_mim]^+^	[C_8_(mim)_2_]^2+^	[choline]^+^	[BTMA]^+^
M–O	*N*	6.0	6.0	6.0	6.0	6.0	6.0	6.0	6.0
	*R* (Å)	2.07	2.07	2.07	2.07	2.05	2.05	2.04	2.05
	σ^2^ (Å^2^)	0.003	0.003	0.003	0.003	0.003	0.003	0.003	0.003
	β	0.4	0.4	0.4	0.4	0.4	0.4	0.4	0.4
									
M–S	*N*	6.0	6.0	6.0	6.0	6.0	6.0	6.0	6.0
	*R* (Å)	3.48	3.48	3.48	3.48	3.46	3.46	3.45	3.46
	σ^2^ (Å^2^)	0.005	0.005	0.005	0.005	0.005	0.005	0.005	0.005
	β	0.0	0.0	0.0	0.0	0.0	0.0	0.0	0.0
									
M–N_1_	*N*	3.0	4.1	3.1	3.0	3.3	3.2	3.3	3.2
	*R* (Å)	4.22	4.28	4.28	4.27	4.26	4.27	4.25	4.25
	σ^2^ (Å^2^)	0.035	0.035	0.035	0.033	0.033	0.030	0.034	0.036
	β	0.0	–0.1	–0.2	–0.2	–0.1	–0.3	–0.1	–0.1
									
M–N_2_	*N*	3.0	1.9	2.9	3.0	2.7	2.8	2.7	2.8
	*R* (Å)	4.45	4.47	4.46	4.47	4.44	4.44	4.44	4.44
	σ^2^ (Å^2^)	0.017	0.013	0.016	0.014	0.014	0.014	0.014	0.014
	β	0.0	0.0	0.0	0.0	0.0	0.0	–0.3	0.0

a M–N_1_ and M–N_2_ refer to the two functions employed to fit the M–N pair distributions.

b Data for Co^2+^ in [C_4_mim][Tf_2_N] taken from ref (48).

**Figure 4:**
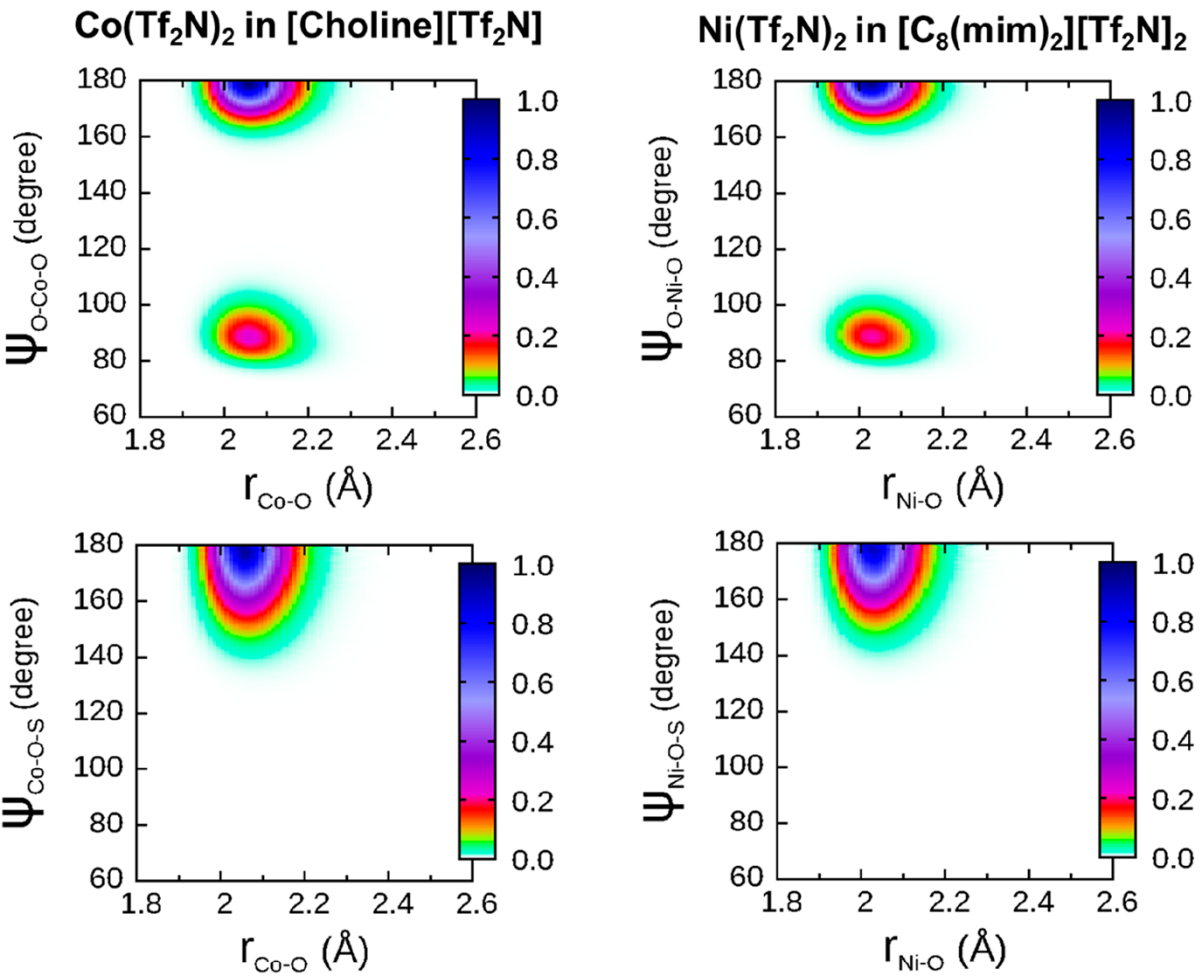
Combined distribution functions (CDFs) between the Co–O distances and the O–Co–O angles as well as between the Co–O distances and the Co–O–S angles calculated from the MD simulation of 0.1 mol L^–1^ Co(Tf_2_N)_2_ in [choline][Tf_2_N] (left panels); CDFs between the Ni–O distances and the O–Ni–O angles as well as between the Ni–O distances and the Ni–O–S angles calculated from the MD simulation of 0.1 mol L^–1^ Ni(Tf_2_N)_2_ in [C_8_(mim)_2_][Tf_2_N]_2_ (right panels). The [Tf_2_N]^−^ oxygen atoms included in the CDFs are inside a 2.60 Å cutoff from the metal ions, while sulfur atoms are those covalently bonded to the selected oxygen atoms. Color-boxes on right sides are relative to the probability of finding the inspected atoms at that distance and angle.

To get further insights into the arrangement of the [Tf_2_N]^−^ ligands around the metal ions, the CDFs between the M–O distances and the O–M–O angles have been calculated, together with those between the M–O distances and the M–O–S angles. Note that CDFs are in general more informative than the commonly employed radial or angular distribution functions alone, as they can provide key-information on the distance-angle correlation.^62^ The CDFs have been reported for Co(Tf_2_N)_2_ in [choline][Tf_2_N] and for Ni(Tf_2_N)_2_ in [C_8_(mim)_2_][Tf_2_N]_2_ as examples (Figure 4). The obtained CDFs show similar features in both systems and well-defined regions of high intensity are found at M–O distances comparable to the average bond lengths reported in Table 1. As concerns the angles, in the O–M–O distributions two peaks are found at 90° and 180°, with this the typical fingerprint of an octahedral coordination geometry. Conversely, the M–O–S distributions show a single feature around 180°. It should be noted that bent M–O–S configurations with bond angles between 135° and 145° were previously found to arise from bidentate binding [Tf_2_N]^−^ anions in the case of the Zn^2+^ ion in the same ILs studied in this work.^46^ The absence of this feature in the CDFs computed for the Ni^2+^ and Co^2+^ cations is therefore a further proof for the monodentate coordination of the bistriflimide ligand toward these ions.

Altogether these results suggest that, in the IL solutions, the Co^2+^ and Ni^2+^ cations are coordinated by six monodentate [Tf_2_N]^−^ ligands forming octahedral complexes (Figure 2B), independently from the IL organic cation. The same coordination was found in case of the Zn^2+^ and Co^2+^ ions in [C_4_mim][Tf_2_N] in previous works.^46,48^ In addition, very small structural differences are observed for the same metal ion when considering different IL cations looking at the structural parameters listed in Table 1. This finding is different from what was obtained for the Zn^2+^ ion, where an equilibrium between the 5-fold [Zn(Tf_2_N)_5_]^3–^ and the 6-fold [Zn(Tf_2_N)_6_]^4–^ species was observed in [C_8_(mim)_2_][Tf_2_N]_2_, [choline][Tf_2_N], and [BTMA][Tf_2_N].^46^ Since both complexes present an octahedral coordination, in the 5-fold one four [Tf_2_N]^−^ anions coordinate the metal in a monodentate fashion, while one [Tf_2_N]^−^ anion behaves as bidentate and assumes a bent M–O–S configuration. Conversely, a pure monodentate [Tf_2_N]^−^ coordination is present in the [Zn(Tf_2_N)_6_]^4^ complex. The percentage of bidentate coordination was observed to increase by considering different organic cations in the order [choline]^+^ > [BTMA]^+^ > [C_8_(mim)_2_]^2+^, while [C_4_mim][Tf_2_N] was the only IL showing a pure monodentate coordination. The total absence of the bidentate coordination in the case of the Co^2+^ and Ni^2+^ ions in all the investigated ILs marks a difference in the nature of the interaction with the [Tf_2_N]^−^ anion between the studied metal ions and the Zn^2+^ case.

### 3.2 Co^2+^ and Ni^2+^ Coordination: XAS Results

#### 3.2.1 [Tf_2_N]^−^ Anion Coordination

**Figure 5:**
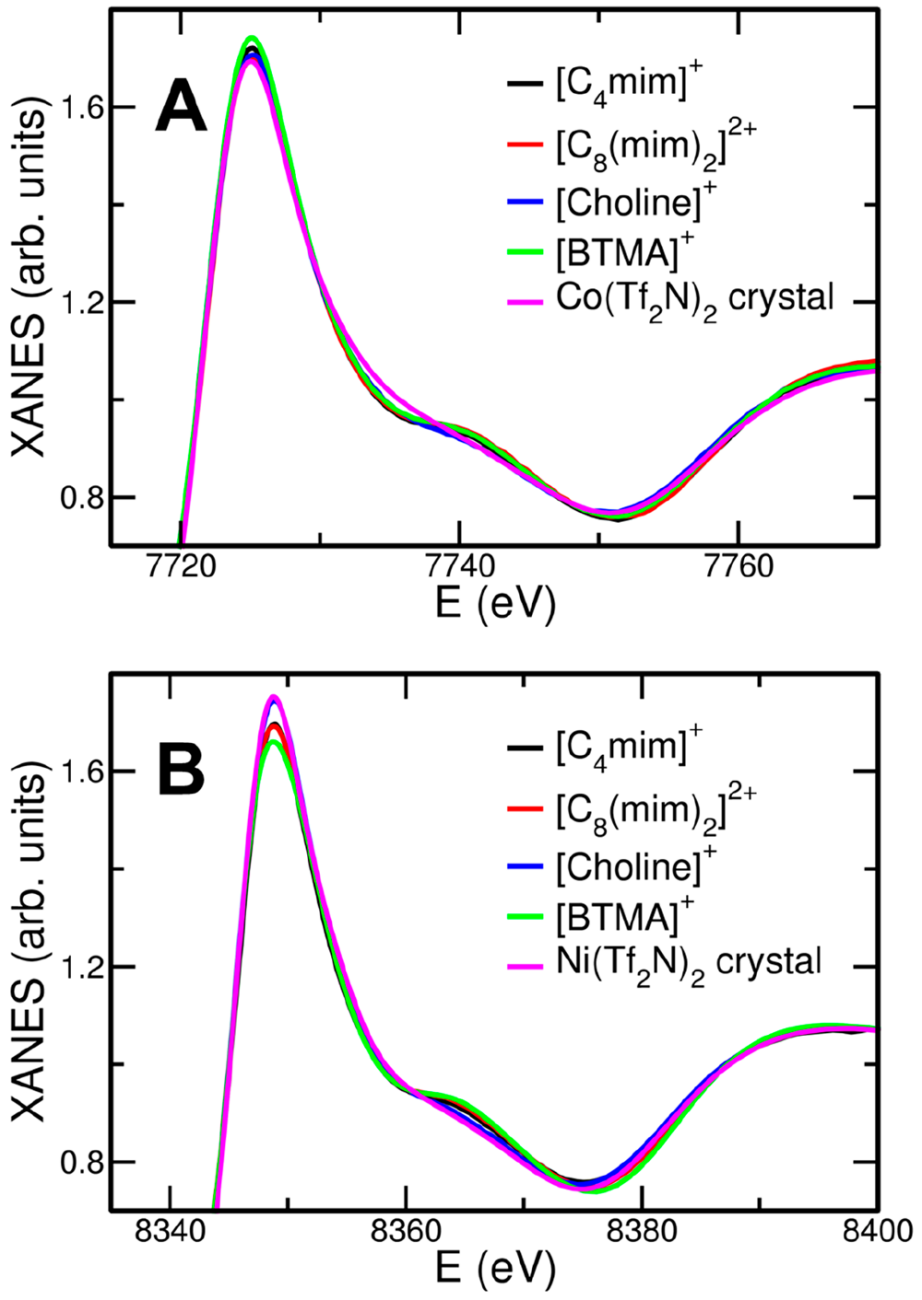
(A) Co K-edge and (B) Ni K-edge XANES experimental spectra of solid Co(Tf_2_N)_2_ and Ni(Tf_2_N)_2_, respectively, compared with the 0.1 mol L^–1^ solutions in ILs carrying the [Tf_2_N]^−^ anion with different organic cations, namely, [C_4_mim]^+^ (black lines), [C_8_(mim)_2_]^2+^ (red lines), [choline]^+^ (blue lines), and [BTMA]^+^ (green lines).

The structural arrangement of the [Tf_2_N]^−^ anion around the Co^2+^ ion in the solid state was determined in previous works.^48,72^ In the crystallographic structure, the cation is coordinated by four [Tf_2_N]^−^ ligands, two of which are monodentate while the other two are bidentate (Figure 2A). Six oxygen atoms are found at an average Co–O distance of 2.07 Å (Table 2), while a bent Co–O–S configuration with an angle of 134° is present both in the mono- and bidentate ligands. Six sulfur atoms are also found at 3.29 Å and four nitrogen atoms at 3.60 Å from the metal (Table 2). A comparison between the XANES (X-ray absorption near edge structure) part of the absorption spectra collected on the crystalline sample and on the Co(Tf_2_N)_2_ salt solutions has been carried out to obtain first qualitative information on the different coordination of the solid state and the solution systems, since it is known that XANES is very sensitive to the three-dimensional displacement of the closest scattering centers.^38,76^ The XANES spectrum of solid Co(Tf_2_N)_2_, together with those of Co(Tf_2_N)_2_ solutions in [C_4_mim][Tf_2_N], [C_8_(mim)_2_][Tf_2_N]_2_, [choline][Tf_2_N], and [BTMA][Tf_2_N] is reported in Figure 5A. As can be observed, the XANES spectrum of the crystal shows small but detectable differences as compared to those of the solutions.^48^ In particular, the IL solutions show the presence of a bump at about 7740 eV that is not observed in the crystal spectrum, thus suggesting that the ligands assume a different structural arrangement in the two cases. A similar behavior is observed in the case of the of solid Ni(Tf_2_N)_2_ and its IL solutions, as shown in Figure 5B, where the spectra of the liquid samples show a bump at about 8370 eV, while this feature is absent in the spectrum of the solid sample. Note that this behavior was already observed when comparing the XANES spectrum of the Zn(Tf_2_N)_2_ salt to the those collected on Zn(Tf_2_N)_2_ solutions in the [C_4_mim][Tf_2_N], [C_8_(mim)_2_][Tf_2_N]_2_, [choline][Tf_2_N], and [BTMA][Tf_2_N] ILs.^46^ Also in that case, the octahedral [Zn(Tf_2_N)_4_]^2–^ species (two mono- and two bidentate [Tf_2_N]^−^) was detected for the solid state.^46,77^ This suggests that, even for the Ni^2+^ ion, the ILs are able to dissolve the metal salt giving rise to a different coordination around the metal with respect to the solid state.

**Table 2:** Coordination Number *N*, Average Distance *R* (Å), Debye–Waller Factor σ^2^ (Å^2^), and Asymmetry Parameter β as Obtained from the EXAFS Data Analysis of the Co(Tf_2_N)_2_ and Ni(Tf_2_N)_2_ Crystals and for their 0.1 mol L^–1^ Solutions in ILs Carrying the [Tf_2_N]^−^ Anion with Different Organic Cations: [C_4_mim]^+^, [C_8_(mim)_2_]^2+^, [choline]^+^, and [BTMA]^+^

		M = Co^2+^					M = Ni^2+^				
path		Co(Tf_2_N)_2_^a^	[C_4_mim]^+^^a^	[C_8_(mim)_2_]^2+^	[choline]^+^	[BTMA]^+^	Ni(Tf_2_N)_2_	[C_4_mim]^+^	[C_8_(mim)_2_]^2+^	[choline]^+^	[BTMA]^+^
M–O	*N*	6	6	6	6	6	6	6	6	6	6
	*R*	2.07(2)	2.08(1)	2.10(2)	2.10(2)	2.09(2)	2.06(2)	2.06(2)	2.07(2)	2.06(2)	2.07(2)
	σ^2^	0.006(2)	0.005(2)	0.005(2)	0.004(2)	0.005(2)	0.004(2)	0.003(2)	0.004(2)	0.004(2)	0.004(2)
	β	0.0(2)	0.0(2)	0.1(2)	0.0(2)	0.1(2)	0.0(2)	0.2(2)	0.2(2)	0.0(2)	0.2(2)
											
M–S	*N*	6	6	6	6	6	6	6	6	6	6
	*R*	3.29(3)	3.49(3)	3.50(3)	3.55(3)	3.53(3)	3.41(3)	3.49(3)	3.52(3)	3.49(3)	3.51(3)
	σ^2^	0.008(2)	0.009(3)	0.010(3)	0.010(3)	0.010(3)	0.010(2)	0.010(3)	0.010(3)	0.010(3)	0.010(3)
	β	0.0(2)	0.0(3)	0.1(3)	0.0(3)	0.0(3)	0.0(2)	0.0(3)	0.0(3)	0.1(3)	0.0(3)
											
M–N	*N*	4	6	6	6	6	6	6	6	6	6
	*R*	3.60(4)	4.39(4)	4.35(4)	4.35(4)	4.40(4)	3.57(4)	4.39(4)	4.40(4)	4.40(4)	4.36(4)
	σ^2^	0.009(2)	0.014(4)	0.015(4)	0.015(4)	0.015(4)	0.007(2)	0.020(4)	0.020(4)	0.017(4)	0.017(4)
	β	0.1(2)	0.1(3)	0.1(3)	0.1(3)	0.1(3)	0.0(4)	0.1(3)	0.1(3)	0.0(3)	0.2(3)

a Data for the Co(Tf_2_N)_2_ crystal and for the Co(Tf_2_N)_2_ 0.1 mol L^–1^ solution in [C_4_mim][Tf_2_N] are taken from ref (48).

#### 3.2.2 EXAFS Experimental Results

**Figure 6:**
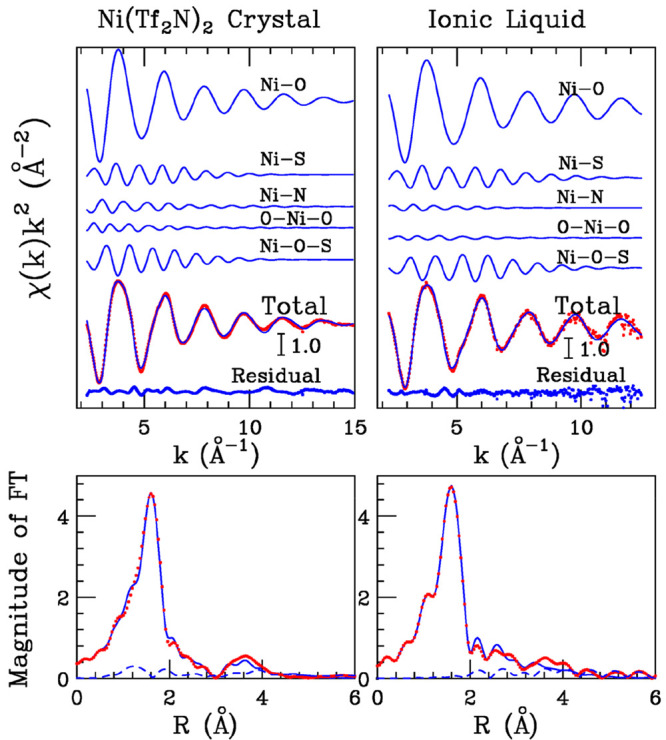
Upper panels: fit of the Ni K-edge EXAFS spectra of Ni(Tf_2_N)_2_ crystal (left panel) and of the 0.1 mol L^–1^ Ni(Tf_2_N)_2_ solution in [C_4_mim](Tf_2_N) (right panel). From the top to the bottom: Ni–O, Ni–S, and Ni–N SS theoretical signals, O–Ni–O and Ni–O–S MS three-body theoretical signals, total theoretical signal (blue line) compared with the experimental spectrum (red dotted line), and the residual curve. Lower panels: nonphase shift corrected Fourier transforms of the experimental data (red dotted line), of the total theoretical signals (blue line) and of the residual curves (blue dashed line).

In order to have definitive proof of the hypothesis suggested by the XANES data and to obtain a description of the short-range structural arrangement around the Ni^2+^ ion in the metallic salt in a more quantitative way, the analysis of the EXAFS spectrum collected on the solid Ni(Tf_2_N)_2_ sample has been carried out. To this end, the coordination previously obtained for the Co(Tf_2_N)_2_ salt^48,72^ has been employed as a starting point (Figure 2A). Two body signals associated with six Ni–O, six Ni–S, and four Ni–N paths have been included, while the MS signals associated with three linear O–Ni–O contributions and six Ni–O–S paths having a bent configuration with an angle of 155° also showed a detectable amplitude. Least-squares fits have been carried out in the *k* range 2.3–14.9 Å^–1^ on the raw spectrum, and the best-fit results are shown in the upper left panel of Figure 6, where the theoretical two- and three-body signals are depicted together with the total theoretical contribution compared with the experimental data. As can be observed, a very good agreement between the theoretical and experimental data is shown, as it is also evident from the corresponding FT spectra shown in the lower-left panel of Figure 6. The FTs have been calculated in the 2.1–14.0 Å^–1^*k* range. The structural parameters obtained after the minimization procedures are listed in Table 2, while the *E*_0_ value resulted to be 3 eV above the first inflection point of the experimental spectrum. From the EXAFS analysis of the Ni(Tf_2_N)_2_ salt, the metal ion has been found to be coordinated by six oxygen atoms at a distance of 2.06 Å, while six sulfur atoms are placed at 3.41 Å and four nitrogen atoms at 3.57 Å. This confirms that the Ni^2+^ ion is coordinated by two mono- and two bidentate [Tf_2_N]^−^ anions forming the [Ni(Tf_2_N)_4_]^2–^ unit (Figure 2A), as previously observed for the Co(Tf_2_N)_2_ and Zn(Tf_2_N)_2_ salts on the basis of EXAFS^46,48^ and crystallographic data.^72,77^

To determine the local structure around the Co^2+^ and Ni^2+^ ions in these systems and to assess the validity of the MD simulations results, the analysis of the EXAFS spectra of the IL solutions has been carried out in a second step of this investigation. To this end, the EXAFS M–O, M–S, and M–N two-body theoretical signals, together with those connected with the M–O–S paths, have been calculated starting from the parameters obtained from the MD simulations (Table 1). Note that in this case the theoretical three-body M–O–S signals have been calculated considering the linear configuration obtained from the analysis of the CDFs as previously discussed (Figure 4). Three O–M–O paths consistent with an octahedral configuration have been also included in the analysis. Least-squares fits of the EXAFS data have been carried out in the *k* = 2.0–13.0 Å^–1^ range, and the best-fit results are shown in the upper right panel of Figure 6 for Ni(Tf_2_N)_2_ in [C_4_mim][Tf_2_N] as an example, while the results for the other IL samples are reported in Figures S2 and S3. In all cases, the agreement between the theoretical and experimental data is very good, and this is confirmed by the FT spectra shown in the lower right panel of Figure 6 (calculated in the *k* range of 2.1–12.0 Å^–1^) and the same is observed for the other IL samples. In all systems, both the Co^2+^ and Ni^2+^ metal ions have been found to be coordinated by six oxygen atoms, and degeneracies of six have been found also for the M–S and M–N paths (Table 2). Therefore, the EXAFS results confirm that in the IL solutions the Co^2+^ and Ni^2+^ ions are mainly coordinated by monodentate bistriflimide ligands having a linear M–O–S geometry, while in the crystal the two ions are coordinated by two bidentate and two monodentate ligands. It is important to note that the EXAFS experimental spectra of the solid samples and IL solutions appear as quite similar (Figure 6 and Figures S2 and S3), at variance of the XANES data (Figure 5). In fact, the XANES region is known to be more sensitive to the MS effects, while the high-energy part of the XAS spectrum is dominated by the first shell SS contribution. This latter is dominated by the six oxygen atoms that coordinate the Co^2+^ and Ni^2+^ ions assuming an octahedral geometry in both the solid state and in the ILs solutions. However, small differences in the amplitude and frequency can be observed that are associated with the slightly different structural organization around the metal cation. In particular, in the case of Zn^2+^, this difference was attributed to the different M–O–S distributions between the solid state and the solutions.^46^

A last point we would like to underline is that the MD simulations were able to reproduce the Ni^2+^ and Co^2+^ ions as octahedrally coordinated by six [Tf_2_N]^−^ monodentate anions, in agreement with the experimental evidence. This is a nontrivial result, since the ILs interaction potential was parametrized to reproduce target liquid bulk properties of the pure solvents.^49–52^ Finally, the EXAFS and MD results show that, for the studied ILs, the organic cation has little or no influence on the spatial arrangement of the coordination shell formed by the [Tf_2_N]^−^ ligands around the Co^2+^ and Ni^2+^ ions, with [M(Tf_2_N)_6_]^4–^ as the dominant species detected in all systems.

## 4 Conclusions

The structural characterization of Co(Tf_2_N)_2_ and Ni(Tf_2_N)_2_ solutions in Tf_2_N-based ILs having different organic cations ([C_4_(mim)]^+^, [C_8_(mim)_2_]^2+^, [choline]^+^, and [BTMA]^+^) has been carried out using an integrated approach combining MD simulations and EXAFS experimental data. Co and Ni K-edge X-ray absorption spectra of crystalline Co(Tf_2_N)_2_ and Ni(Tf_2_N)_2_ and of solutions of these salts in the ILs have been compared and analyzed. The EXAFS data analysis of the IL solutions has been carried out starting from the structural description of the first solvation shell provided by MD simulations. The good agreement between theory and experiment allowed us to assess the reliability of the MD structural results for all the investigated systems. After dissolution of the cobalt and nickel salt in the IL solvents, a different structural arrangement around the metal cations is found as compared to that of the salt crystallographic structure. In particular, the Co^2+^ and Ni^2+^ ions are coordinated by two bidentate and two monodentate bistriflimide ligands in the crystal. Conversely, in the IL solutions, both the Co^2+^ and Ni^2+^ ions are coordinated by six monodentate [Tf_2_N]^−^ ligands with a linear M–O–S configuration, forming an octahedral complex. Moreover, the nature of the organic cation has been found to have little or no influence on the overall spatial arrangement of the [Tf_2_N]^−^ anions in the metal first solvation shell.

## Supporting Information Available

Additional characterization data and spectra (PDF)
