# Supplementary material for: Coordination of the Co2+ and Ni2+ Ions in Tf2N– Based Ionic Liquids: A Combined X-ray Absorption and Molecular Dynamics Study
Source: J Phys Chem B. 2021 Jun 10;125(24):6639–48. doi: 10.1021/acs.jpcb.1c03395 (PMC8279557; doi:10.1021/acs.jpcb.1c03395)
Supplement: Supplementary file 1 — jp1c03395_si_001.pdf [file jp1c03395_si_001.pdf]

On the Coordination of the Co^{2+} and Ni^{2+} Ions in Tf_2N^- Based Ionic Liquids: a Combined X-ray Absorption and Molecular Dynamics Study

Matteo Busato,[†,‡] Andrea Lapi,[†] Paola D'Angelo,[*,†] Andrea Melchior[*,‡]

[†]Dipartimento di Chimica, Università di Roma "La Sapienza", P.le A. Moro 5, 00185 Roma, Italy

[‡]DPIA, Laboratorio di Scienze e Tecnologie Chimiche, Università di Udine, Via del Cotonificio 108, 33100 Udine, Italy

*Corresponding author(s):

p.dangelo@uniroma1.it

andrea.melchior@uniud.it

Supporting Information

Table S1. Number of ions and box dimensions of the simulated MD systems.

	Co^{2+}				Ni^{2+}			
System	Co^{2+}	IL cations	$[Tf_2N]^-$	Box edge (Å)	Ni^{2+}	IL cations	$[Tf_2N]^-$	Box edge (Å)
$[C_4mim][Tf_2N]$	5	170	180	44.15	5	170	180	44.14
$[C_8(mim)_2][Tf_2N]_2$	5	90	190	44.30	5	90	190	44.27
$[Choline][Tf_2N]$	5	195	205	44.22	5	195	205	44.20
$[BTMA][Tf_2N]$	5	175	185	44.37	5	175	185	44.21

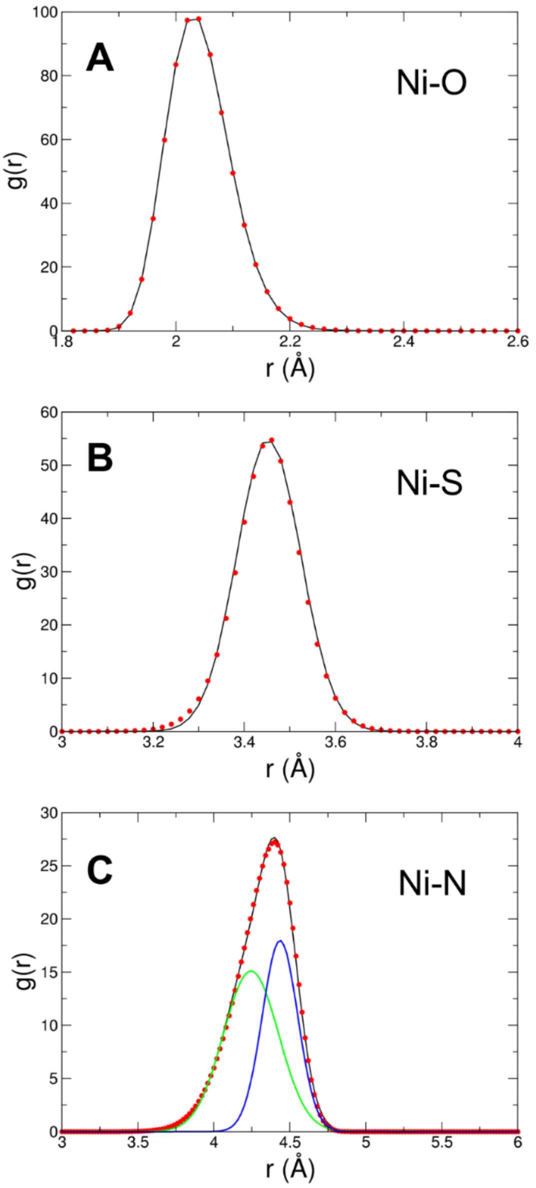

Figure S1. A) Ni-O, B) Ni-S and C) Ni-N *g(r)*'s obtained from the MD simulation for the calculation of the structural properties of the 0.1 mol L^{-1} $Ni(Tf_2N)_2$ solution in $[C_4mim][Tf_2N]$ (red dotted lines) compared with the Γ-like functions obtained from the fitting procedures (solid black lines). For the Ni-N distribution, the two partial curves employed for the fit are also showed (green and blue lines).

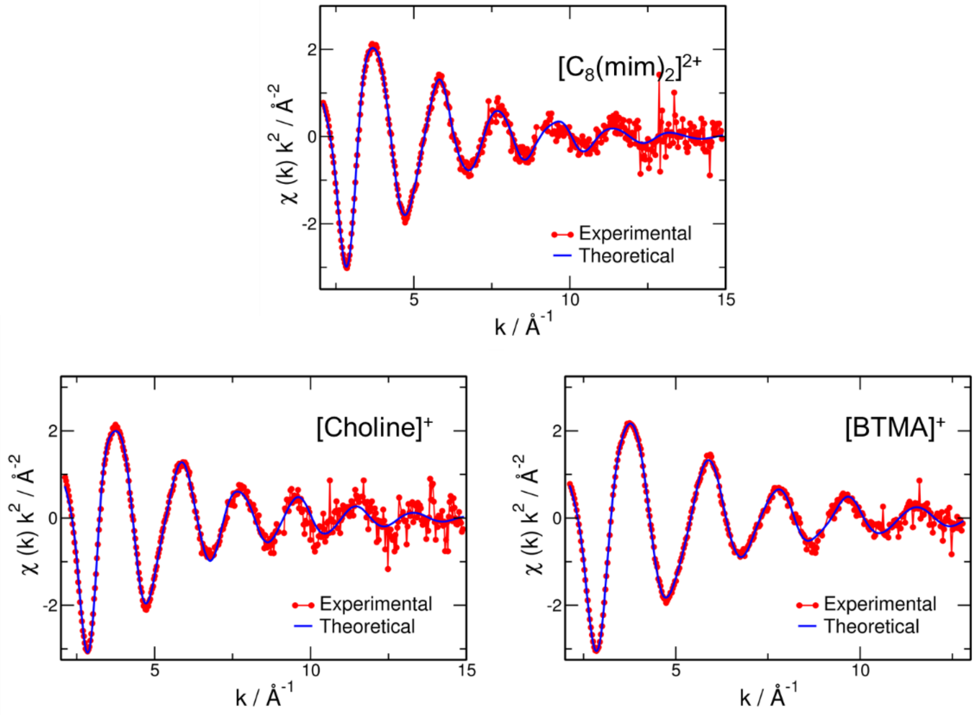

Figure S2. Fit of Co K-edge EXAFS experimental spectra (red dotted lines) of $Co(Tf_2N)_2$ 0.1 mol L^{-1} solutions in Tf_2N-based ILs with different organic cations: $[C_8(mim)_2]^{2+}$, $[Choline]^+$ and $[BTMA]^+$. Total theoretical signals are shown by blue lines.

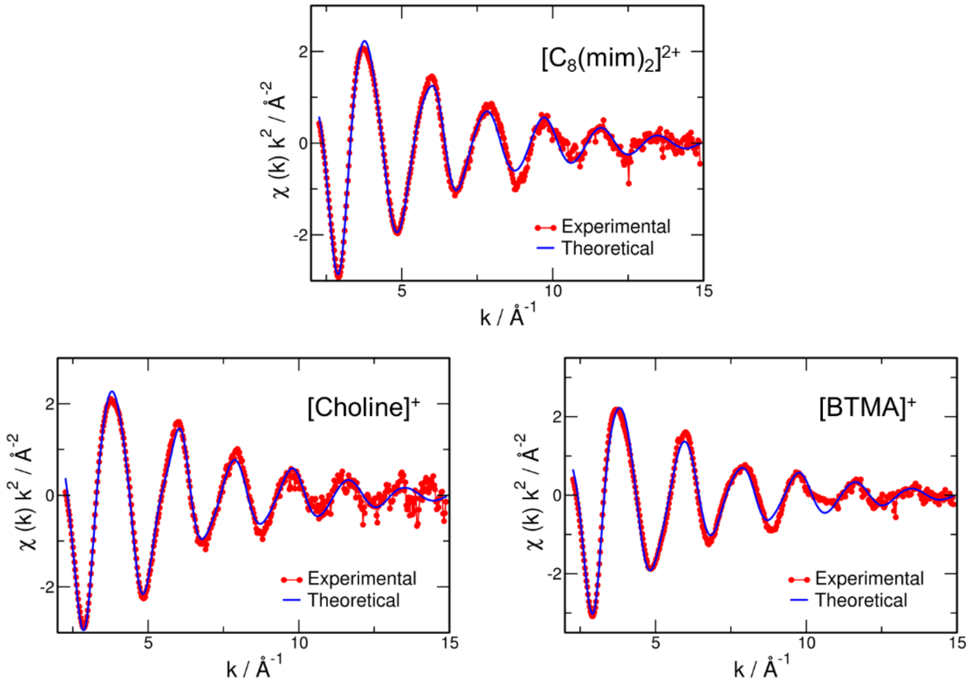

Figure S3. Fit of Ni K-edge EXAFS experimental spectra (red dotted lines) of $Ni(Tf_2N)_2$ 0.1 mol L^{-1} solutions in Tf_2N-based ILs with different organic cations: $[C_8(mim)_2]^{2+}$, $[Choline]^+$ and $[BTMA]^+$. Total theoretical signals are shown by blue lines.
